# Serum magnesium is associated with osteoporosis risk in postmenopausal women: a retrospective study and risk-prediction model

**DOI:** 10.3389/fmed.2026.1770830

**Published:** 2026-03-03

**Authors:** Dongliang Shi, Xiaoyu Li, Nuonan Men, Bowen Ren, Yunfeng Ma, Lisha Wang, Zhongbo Zhang, Kaiguang He, Xuzhao Du, Jili Wang

**Affiliations:** 1 The Second Affiliated Hospital of Henan University of Chinese Medicine, Zhengzhou, China; 2 Henan Provincial Hospital of Traditional Chinese Medicine, Zhengzhou, China; 3 Henan University of Chinese Medicine, Zhengzhou, China; 4 The Third Affiliated Hospital of Henan University of Chinese Medicine, Zhengzhou, China

**Keywords:** bone mineral density, magnesium, osteoporosis, postmenopausal women, risk prediction model

## Abstract

**Objective:**

To investigate the association between serum magnesium (Mg) levels and osteoporosis (OP)/osteoporotic fractures in postmenopausal women, and to develop a multi-parameter model for predicting OP risk.

**Methods:**

This retrospective study included 496 hospitalized postmenopausal women from January 1, 2021 to June 30, 2025. Patients were categorized into the OP group (T-score ≤ −2.5) and the non-OP group (*T*-score>−2.5). Clinical characteristics, bone mineral density (BMD), and laboratory indices were compared between groups. and additional comparisons were performed between patients with and without osteoporotic fractures. Receiver operating characteristic (ROC) curves were used to evaluate the discrimination of Mg and phosphorus (P) for OP. Binary logistic regression was performed with OP as the outcome. Variables selected by least absolute shrinkage and selection operator (LASSO) regression were used to construct a nomogram, which was evaluated using calibration curves and decision curve analysis (DCA).

**Results:**

Among 496 participants, 208 were classified as non-OP and 288 as OP. Osteoporotic fractures were identified in 31 patients, while 465 had no osteoporotic fractures. patients with OP were older, had lower body mass index (BMI), lower BMD, and lower serum Mg and P levels. ROC analysis showed modest discrimination for Mg (AUC 0.602, 95% CI 0.551–0.652), with an optimal cut-off of 0.845 mmol/L. After adjustment for potential confounders, Mg ≤ 0.845 mmol/L was independently associated with higher odds of OP (OR 1.940, 95% CI 1.312–2.868; *P* < 0.05). β-C-terminal telopeptide of type I collagen (β-CTx) (OR 2.229, 95% CI 1.034–4.809), age (OR 1.067, 95% CI 1.042–1.092), and BMI (OR 0.894, 95% CI 0.846–0.945) were also independently associated with OP (all *P* < 0.05). Patients with osteoporotic fractures were older and had lower BMD and lower Mg and P levels than those without fractures. A nomogram incorporating BMI, age, Mg, P, β-CTx, and procollagen type I N-terminal propeptide (P1NP) showed acceptable calibration and potential net clinical benefit.

**Conclusion:**

Lower serum Mg levels were associated with OP and osteoporotic fractures in hospitalized postmenopausal women. Mg may support OP risk stratification, and a multi-parameter model integrating clinical and bone metabolism–related markers may further improve risk assessment.

## 1 Background

Osteoporosis (OP) is a systemic skeletal disorder characterized by low bone mass, microarchitectural deterioration, and increased bone fragility, and it is a major contributor to disability and mortality in older adults (1). With ongoing population aging, the incidence of OP and fragility fractures continues to increase, imposing substantial burdens on quality of life and healthcare systems (2, 3).

Dual-energy X-ray absorptiometry (DXA)–derived bone mineral density (BMD) remains the clinical standard for diagnosing OP (4). Bone turnover markers (BTMs), including osteocalcin (OCN), β-C-telopeptide of type I collagen (β-CTx), and procollagen type I N-terminal propeptide (P1NP), are useful for monitoring skeletal remodeling and may provide prognostic information on fracture risk (5). However, BTMs testing is not uniformly implemented in routine practice, partly due to issues such as cost and analytical variability. In contrast, routinely collected serum electrolytes—including magnesium (Mg) and phosphorus (P)—are inexpensive and widely available in hospitalized patients. Emerging evidence links lower serum Mg and altered serum phosphate levels to higher fracture risk, suggesting a potential role in risk stratification that remains insufficiently translated into clinical algorithms (6–8).

Mg is the fourth most abundant mineral in the human body, with approximately 50–60% stored in bone. It contributes to bone mineralization and functions as a cofactor for numerous enzymatic reactions and adenosine triphosphate (ATP)-dependent processes (9). Mg homeostasis is primarily regulated by intestinal absorption, skeletal storage, and renal excretion (10). Experimental and clinical evidence indicates that Mg deficiency may adversely affect bone metabolism through multiple pathways, including impaired parathyroid hormone (PTH) secretion and vitamin D metabolism, disruption of calcium (Ca) homeostasis, altered hydroxyapatite crystal structure, and activation of pro-inflammatory signaling, thereby contributing to bone loss and fragility (9, 11, 12). Although Ca and Phosphorus (P) have been extensively studied in relation to OP, evidence regarding serum Mg and OP or fragility fractures remains limited and sometimes inconsistent (13). Therefore, this study aimed to investigate the association between serum Mg levels and OP, as well as osteoporotic fractures, in postmenopausal women.

## 2 Materials and methods

### 2.1 Ethical approval

The study protocol was approved by the Ethics Committee of Henan Provincial Hospital of Traditional Chinese Medicine (The Second Affiliated Hospital of Henan University of Chinese Medicine) (approval No. HNSZYYWZ-20250925106).

### 2.2 Study subjects

This retrospective study included postmenopausal women hospitalized in the Department of Orthopedics at Henan Provincial Hospital of Traditional Chinese Medicine between January 1, 2021 and June 30, 2025. The inclusion criteria were: (1) postmenopausal women aged ≥ 50 years; (2) completion of DXA scans of the lumbar spine (L1–L4) and hip during hospitalization; (3) available laboratory results for serum Mg, P, and other relevant indices. Exclusion criteria were: (1) severe hepatic or renal insufficiency; (2) malignancy or metastatic bone disease; (3) other metabolic bone diseases (e.g., hyperparathyroidism, osteomalacia, and Paget’s disease of bone); (4) confirmed long-term use of medications that substantially affect bone metabolism (e.g., long-term high-dose glucocorticoids, high-dose antiresorptive agents, or anabolic therapies). Patients were divided into two groups based on T-score: the OP group (T-score ≤ −2.5) and Non-OP group, (*T*-score > −2.5). The lowest T-score obtained from either the lumbar spine or the hip was used. Patients were future divided into the osteoporotic fracture (OP-fracture) and the non-osteoporotic fracture (Non OP-fracture) group. Osteoporotic fracture was defined as a low-trauma fracture (e.g., a fall from standing height) occurring at typical osteoporotic sites (hip, vertebrae, distal radius, etc.) and confirmed by radiography or other imaging modalities. Fractures resulting from high-energy trauma were excluded. The fracture status reflected that there was a clear history of fracture before admission, and the sites were hip, distal radius, and vertebral body.

### 2.3 Data collection

Demographic characteristics were collected, including age (years), height (cm), and weight (kg). Body mass index (BMI, kg/m^2^) was calculated as calculated as weight (kg) divided by height squared (m^2^). BMD (g/cm^2^) was measured at the lumbar spine (L1–L4), femoral neck, Ward’s triangle, and total hip using DXA (Hologic Discovery Wi, model 010-0575, United States). Fasting venous blood samples were collected in the morning. Serum electrolytes—including potassium (K, 3.5–5.3 mmol/L), sodium (Na, 137–147 mmol/L), chloride (Cl, 99–110 mmol/L), Ca (2.11–2.52 mmol/L), Mg (0.7–1.10 mmol/L), and *P* (0.85–1.51 mmol/L)—were measured using an automated biochemical analyzer (Abbott ARCHITECT c16000, United States). BTMs were quantified using electrochemiluminescence immunoassay (ECLIA) on a Roche cobas e602 platform (Switzerland), including OCN (postmenopausal: 15–46 ng/mL), β-CTx (postmenopausal: < 1.008 pg/mL), and P1NP (postmenopausal: 20.25–76.31 pg/mL).

### 2.4 Statistical analysis

Continuous variables are presented as mean ± standard deviation (SD) and compared using the independent-samples *t* test. Receiver operating characteristic (ROC) curves were used to evaluate the discriminative performance of serum Mg and P for OP, and the area under the curve (AUC) with 95% confidence interval (CI) was calculated. Based on the ROC analysis, serum Mg was dichotomized using the optimal cutoff (0.845 mmol/L) as ≤ 0.845 mmol/L vs. > 0.845 mmol/L. Binary logistic regression was performed with OP as the dependent variable and Mg category, *P*, OCN, β-CTx, P1NP, age, and BMI as independent variables. odds ratio (OR) with 95% CI was reported. To further support the logistic regression findings, least absolute shrinkage and selection operator (LASSO) regression was used to select predictors with non-zero coefficients for multivariable model development. Subsequently, a multivariable logistic regression model was built using the predictors selected by LASSO. Calibration curves and decision curve analysis (DCA) were used to assess calibration and clinical utility of the risk-prediction nomogram. All statistical analyses were performed using SPSS version 26.0 (IBM Corp., Armonk, NY, United States). *P* < 0.05 was considered statistically significant.

## 3 Results

### 3.1 Comparison of clinical characteristics and laboratory parameters between the OP and non-OP groups

A total of 496 patients were included, with 208 classified as Non-OP and 288 as OP. Compared with the Non-OP group, patients in the OP group were significantly older and had a lower BMI (both *P* < 0.05). Lumbar spine and hip-related BMD values were significantly lower in the OP group (all *P* < 0.05). Serum Mg and P levels were also lower in the OP group (*P* < 0.05). No significant between-group differences were observed in serum Ca, Na, K, or Cl, or in OCN, β-CTx, and P1NP (all *P* > 0.05) (Table 1).

**TABLE 1:** Clinical and laboratory characteristics between Non-OP group and OP group.

Variables	Non-OP group	OP group	*P*-value
Number	208	288	
Age (years)	62.15 ± 8.56	66.27 ± 8.56	*P* < 0.001
Weight (kg)	65.39 ± 9.59	60.84 ± 9.02	*P* < 0.001
Height (cm)	158.47 ± 5.95	156.11 ± 6.03	*P* < 0.001
BMI (kg/m^2^)	26.02 ± 3.43	24.93 ± 3.63	*P* < 0.001
L1 BMD (g/cm^2^)	0.89 ± 0.12	0.75 ± 0.21	*P* < 0.001
L2 BMD (g/cm^2^)	0.95 ± 0.16	0.75 ± 0.15	*P* < 0.001
L3 BMD (g/cm^2^)	1.01 ± 0.20	0.80 ± 0.13	*P* < 0.001
L4 BMD (g/cm^2^)	1.02 ± 0.19	0.82 ± 0.18	*P* < 0.001
L1-4 BMD (g/cm^2^)	0.97 ± 0.16	0.78 ± 0.13	*P* < 0.001
Femoral neck BMD (g/cm^2^)	0.75 ± 0.13	0.60 ± 0.12	*P* < 0.001
WORD BMD (g/cm^2^)	0.66 ± 0.20	0.43 ± 0.16	*P* < 0.001
Hip BMD (g/cm^2^)	0.89 ± 0.12	0.71 ± 0.13	*P* < 0.001
OCN (ng/mL)	18.73 ± 7.08	19.15 ± 8.35	0.542
β-CTx (pg/mL)	0.47 ± 0.23	0.50 ± 0.27	0.286
P1NP (pg/mL)	54.08 ± 22.96	57.00 ± 39.81	0.343
K (mmol/L)	4.07 ± 0.36	4.04 ± 0.31	0.391
Na (mmol/L)	140.75 ± 2.28	140.95 ± 2.34	0.331
Cl (mmol/L)	105.72 ± 2.50	105.82 ± 2.79	0.664
Ca (mmol/L)	2.33 ± 0.11	2.34 ± 0.12	0.730
Mg (mmol/L)	0.87 ± 0.06	0.84 ± 0.06	*P* < 0.001
P (mmol/L)	1.14 ± 0.14	1.12 ± 0.14	0.043

OP, osteoporosis; BMI, body mass index; BMD, bone mineral density; OCN, osteocalcin; β-CTx,β-collagen C-telopeptide; P1NP, total type I collagen amino-terminal propeptide; K, potassium; Na, sodium; Cl, chloride; Ca, calcium; Mg, magnesium; P, phosphorus. *P* < 0.05 was considered statistically significant.

### 3.2 Independent factors associated with osteoporosis

ROC analysis indicated modest discrimination for Mg (AUC = 0.602, 95% CI 0.551–0.652; *P* < 0.05), with an optimal cutoff of 0.845 mmol/L (sensitivity 67.3%; specificity 47.6%). Serum P showed limited discrimination (AUC = 0.548, 95% CI 0.497–0.600; *P* > 0.05) (Table 2).

**TABLE 2:** Sensitivity analysis of laboratory with osteoporosis.

Variables	AUC	95%CI	*P*-value	Sensitivity	Specificity	Threshold
Mg	0.602	0.551–0.652	*P* < 0.001	0.673	0.476	0.845 mmol/L
P	0.548	0.497–0.600	0.067	0.332	0.774	1.215 mmol/L

Mg, magnesium; P, phosphorus; AUC, area under the curve; CI, confidence interval. *P* < 0.05 was considered statistically significant.

Binary logistic regression was performed to examine the factors influencing OP. In Model 1 (unadjusted): low Mg (≤ 0.845 mmol/L) was associated with higher odds of OP (OR = 1.940, 95% CI, 1.312–2.868; *P* < 0.05). Model 2 (adjusted): after adjustment for P, OCN, β-CTx, P1NP, age, and BMI, low Mg remained independently associated with OP (OR = 1.940, 95% CI, 1.312–2.868; *P* < 0.05), β-CTx was positively associated with OP (OR = 2.229, 95% CI, 1.034–4.809; *P* < 0.05), whereas BMI was inversely associated (OR = 0.894, 95% CI, 0.846–0.945; *P* < 0.05) (Table 3).

**TABLE 3:** Binary logistic regression analysis results of factors of osteoporosis.

Variables	OR	95%	*P-*value
Model 1			
Mg ≤ 0.845 (mmol/L)	1.868	1.289–2.706	0.001
Model 2			
Mg ≤ 0.845 (mmol/L)	1.940	1.312–2.868	0.001
β-CTx	2.229	1.034–4.809	0.041
Age	1.067	1.042–1.092	*P* < 0.001
BMI	0.894	0.846–0.945	*P* < 0.001

Model 1 Mg; Model 2 Mg, P, OCN, β-CTx, PINP, Age, BMI; Mg, magnesium; β-CTx, β-collagen C-telopeptide; BMI, body mass index. *P* < 0.05 was considered statistically significant.

### 3.3 LASSO regression analysis

**FIGURE 1:**
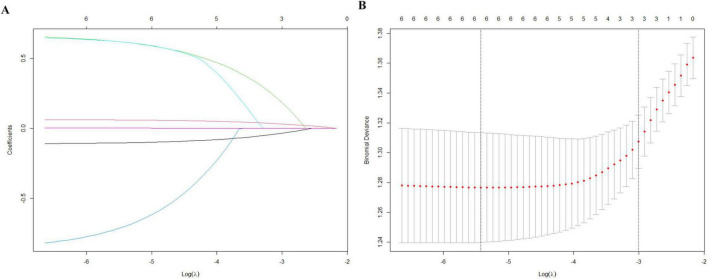
Feature selection and cross-validation for postmenopausal osteoporosis using LASSO regression. **(A)** LASSO coefficient profiles of the six candidate predictors as a function of log(λ). **(B)** Ten-fold cross-validation for selecting the optimal penalty parameter (λ); the value of λ corresponding to the minimum cross-validated error was chosen.

**FIGURE 2:**
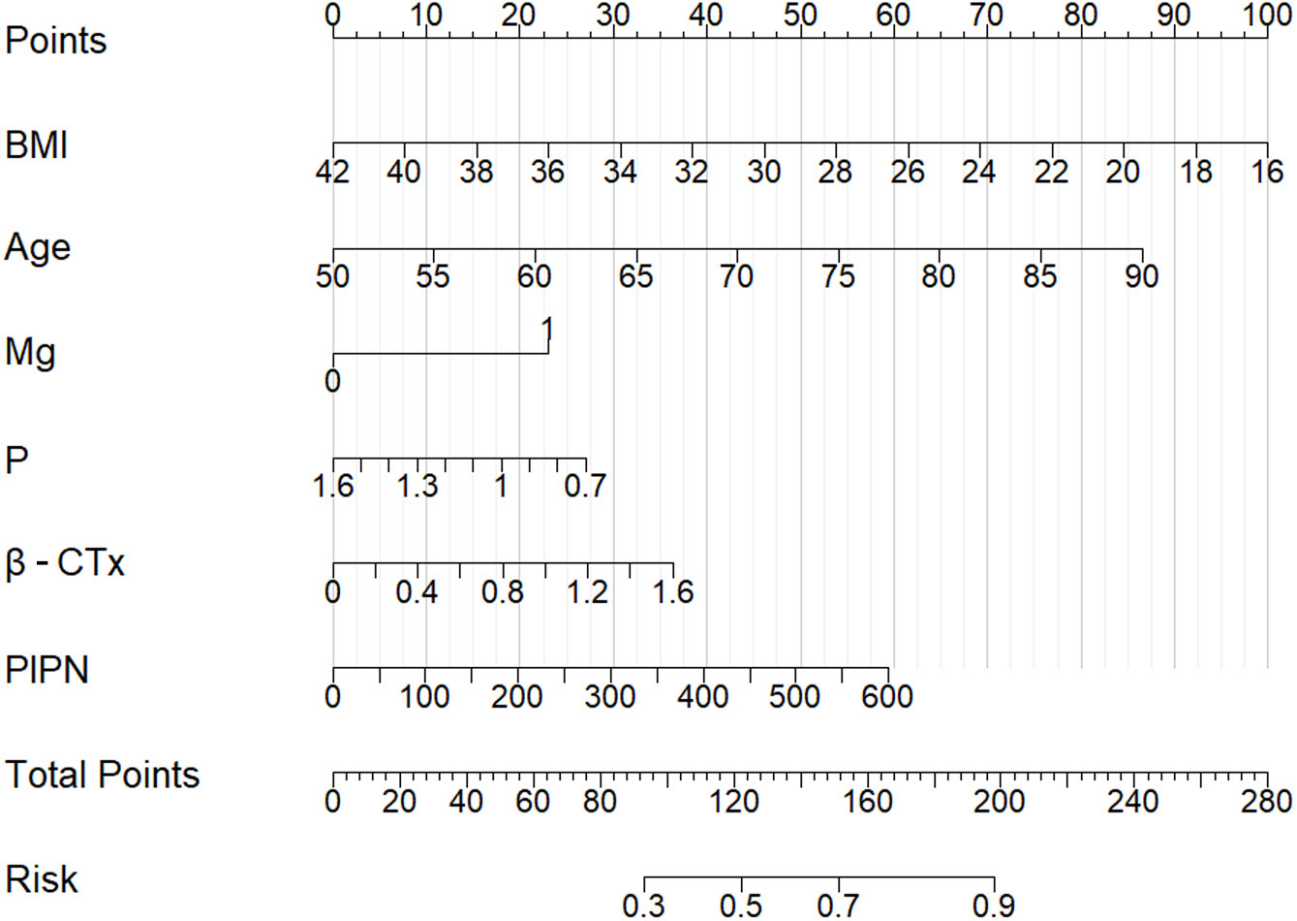
Nomogram for predicting the risk of postmenopausal osteoporosis. The multivariable model incorporates body mass index (BMI), age, serum magnesium (Mg), serum phosphorus (P), β-cross-linked C-terminal telopeptide of type I collagen (β-CTx), and procollagen type I N-terminal propeptide (P1NP).

**FIGURE 3:**
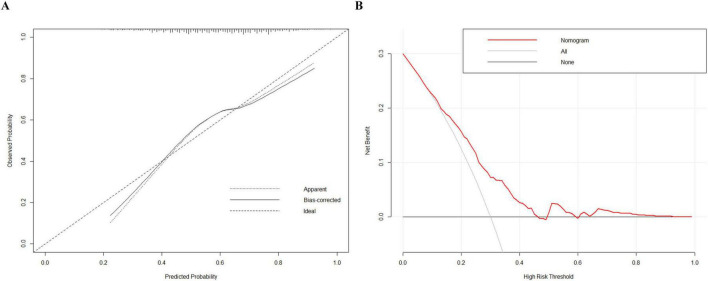
Calibration and clinical utility of the nomogram for postmenopausal osteoporosis risk. **(A)** Calibration plot comparing predicted and observed probabilities of osteoporosis in the study cohort. **(B)** Decision curve analysis (DCA) evaluating the net benefit of the nomogram across a range of threshold probabilities.

LASSO regression selected six predictors with non-zero coefficients: BMI, age, Mg, P, β-CTx, and P1NP (Figure 1). These variables were incorporated into a multivariable model and visualized as a nomogram (Figure 2). The calibration plot demonstrated overall agreement between predicted and observed probabilities, with a slight deviation mainly in the higher predicted probability range. We further quantified the calibration status using the Hosmer-Lemeshow goodness-of-fit test, which revealed a slight inadequacy in calibration (*P* = 0.041). The bias-corrected curve closely followed the ideal line across most of the prediction range (Figure 3A). The DCA demonstrates that this nomogram exhibits potential clinical net benefit across a broad range of threshold probabilities (Figure 3B).

### 3.4 Comparison between the non-fracture and osteoporotic fracture groups

Among the 496 patients, 31 had osteoporotic fractures and 465 were without osteoporotic fractures (Table 4). Patients with osteoporotic fractures were older and had significantly lower BMD at lumbar and hip compared with patients with Non OP-fracture (all *P* < 0.05). Compared with patients with Non OP-fracture, serum Mg, P, and β-CTx levels were also lower in the OP-fracture group (*P* < 0.05).

**TABLE 4:** Clinical and laboratory characteristics between non-fracture group and OP-fracture.

Variables	Non OP-fracture	OP-fracture	*P*-value
Number	465	31	
Age (years)	64.20 ± 8.69	69.61 ± 8.80	0.001
Weight (kg)	62.81 ± 9.54	60.31 ± 9.48	0.151
Height (cm)	157.19 ± 6.12	155.82 ± 5.78	0.224
BMI (kg/m^2^)	25.42 ± 3.58	24.86 ± 3.74	0.386
L1 BMD (g/cm^2^)	0.80 ± 0.18	0.86 ± 0.33	0.337
L2 BMD (g/cm^2^)	0.84 ± 0.17	0.81 ± 0.34	0.606
L3 BMD (g/cm^2^)	0.89 ± 0.19	0.83 ± 0.21	0.102
L4 BMD (g/cm^2^)	0.91 ± 0.21	0.83 ± 0.20	0.043
L1-4 BMD (g/cm^2^)	0.86 ± 0.17	0.84 ± 0.22	0.422
Femoral neck BMD (g/cm^2^)	0.67 ± 0.15	0.60 ± 0.11	0.007
WORD BMD (g/cm^2^)	0.54 ± 0.21	0.42 ± 0.17	0.004
Hip BMD (g/cm^2^)	0.79 ± 0.15	0.70 ± 0.14	0.002
OCN (ng/mL)	19.14 ± 7.82	16.49 ± 7.83	0.068
β-CTx (pg/mL)	0.50 ± 0.26	0.38 ± 0.21	0.017
P1NP (pg/mL)	56.31 ± 34.26	47.77 ± 24.77	0.173
K (mmol/L)	4.06 ± 0.33	4.04 ± 0.34	0.788
Na (mmol/L)	140.91 ± 2.31	140.32 ± 2.30	0.175
Cl (mmol/L)	105.80 ± 2.64	105.39 ± 3.19	0.400
Ca (mmol/L)	2.34 ± 0.11	2.32 ± 0.14	0.606
Mg (mmol/L)	0.86 ± 0.06	0.83 ± 0.07	0.035
P (mmol/L)	1.14 ± 0.14	1.08 ± 0.13	0.036

BMI, body mass index; BMD, bone mineral density; OCN, osteocalcin; β-CTx, β-collagen C-telopeptide; P1NP, total type I collagen amino-terminal propeptide; K, potassium; Na, sodium; Cl, chloride; Ca, calcium; Mg, magnesium; P, phosphorus. *P* < 0.05 was considered statistically significant.

## 4 Discussion

In this retrospective study, we demonstrated that lower serum Mg levels were associated with OP and osteoporotic fractures in hospitalized postmenopausal women. These findings are consistent with previous epidemiological and clinical studies suggesting that inadequate Mg status may contribute to reduced bone mass and increased skeletal fragility (11, 13, 14). Although the discriminative ability of serum Mg alone for identifying OP was modest (AUC ≈ 0.60), Mg is routinely measured, inexpensive, and widely available in clinical practice. Therefore, serum Mg may still provide clinically relevant information as an adjunctive biomarker, particularly when DXA or BTMs testing is unavailable or cannot be performed in a timely manner.

We further observed that patients with osteoporotic fractures had significantly lower serum Mg levels than those without fractures. This finding aligns with evidence from prospective cohort studies and meta-analyses reporting an inverse association between Mg intake or circulating Mg levels and fracture risk (6, 15). In large population-based cohorts, lower dietary or serum Mg has been associated with an increased incidence of hip and total fractures, supporting a potential role of Mg in maintaining bone strength and structural integrity (16). Our results extend these observations to a hospitalized postmenopausal population, suggesting that Mg deficiency may be relevant not only for bone density but also for fracture susceptibility.

The biological mechanisms linking Mg deficiency to OP are multifactorial. First, adequate Mg is essential for normal PTH secretion and target tissue responsiveness; Mg deficiency may impair PTH release or induce PTH resistance, resulting in reduced synthesis of 1,25-dihydroxyvitamin D and decreased intestinal calcium absorption, thereby compromising bone mineralization (11, 17–19). Second, Mg is an integral component of bone mineral and influences the size, structure, and organization of hydroxyapatite crystals; insufficient Mg may lead to abnormal crystal formation, reduced bone stiffness, and increased fragility (20, 21). Third, Mg deficiency has been shown to promote low-grade inflammation, increase the production of pro-inflammatory cytokines such as tumor necrosis factor-alpha (TNF-α) and interleukin-1β (IL-1β), and disrupt the receptor activator of nuclear factor-κB ligand (RANKL/OPG) balance, thereby enhancing osteoclast activity and accelerating bone resorption (22, 23). Finally, Mg plays a role in regulating Ca and P transport and intracellular signaling pathways; disturbances in Mg homeostasis may therefore indirectly affect overall bone remodeling (24).

In the present study, no significant differences in OCN, P1NP, or β-CTx were observed between the OP and Non-OP groups in univariate analyses. However, β-CTx emerged as an independent factor associated with OP in multivariable logistic regression, while β-CTx levels were lower in the osteoporotic fracture group than in the non-fracture group. This apparent inconsistency is likely attributable to age-related heterogeneity in bone turnover. In the univariate analysis, the OP group likely comprised a mixture of high-turnover OP, characterized by increased osteoclast activity, and low-turnover OP, characterized by reduced activities of both osteoclasts and osteoblasts. Such heterogeneity may have attenuated the overall between-group difference, resulting in comparable β-CTx levels—an indicator of osteoclast activity—across groups. In contrast, binary logistic regression analysis, after adjustment for confounding variables, particularly age, revealed an independent association between β-CTx and OP. Moreover, the fracture group was significantly older, and most cases likely represented postmenopausal low-turnover OP. This age-dependent shift in bone turnover pattern may explain the significantly lower β-CTx levels observed in the between-group comparison (25). In addition, BTMs are influenced by multiple factors, such as renal function, circadian variation, and medication exposure, which may not have been fully accounted for in this retrospective analysis (26). These findings highlight the need to interpret BTMs within a comprehensive clinical context rather than in isolation.

An important practical implication of our findings is that Mg status is potentially modifiable. Epidemiological studies indicate that dietary Mg intake is frequently inadequate, and subclinical Mg deficiency is common, particularly among older adults (27, 28). In our study, a serum Mg level ≤ 0.845 mmol/L was independently associated with OP and was also lower in patients with osteoporotic fractures. Clinically, this suggests that attention should be paid to Mg levels when reviewing routine electrolyte panels in postmenopausal women. Although causality cannot be inferred, optimizing dietary Mg intake or correcting Mg deficiency through supplementation may represent a low-cost and accessible strategy to support bone health. Previous studies have linked low Mg status to adverse alterations in bone metabolism, providing biological plausibility for such interventions (14). However, well-designed prospective cohort studies and randomized controlled trials are required to determine whether Mg supplementation can effectively reduce the risk of osteoporosis and fragility fractures (29, 30).

Several limitations of this study should be acknowledged. First, the single-center retrospective design may introduce selection bias and precludes causal inference. Second, important confounding factors—including dietary Mg intake, vitamin D status, PTH levels, and detailed medication history—were not systematically collected. Mg is essential for PTH secretion and vitamin D metabolism. Theoretically, OP patients in this study will have a reduction in PTH and vitamin D levels. However, the lack of data leads to the lack of solid basis for the mechanism of magnesium induced OP, which constitutes a major limitation for this study, and the follow-up study will be improved. Third, serum Mg was measured at a single time point and may not accurately reflect long-term Mg status or intracellular Mg concentrations. Finally, the number of patients with osteoporotic fractures was relatively small, and fracture-related analyses should therefore be interpreted as exploratory.

## 5 Conclusion

In conclusion, our results suggest that BMI, age, Mg, P, β-CTx, and P1NP may function as predictive indicators for OP and may provide potential clinical net benefits. In particular, decreased serum Mg levels were associated with an increased risk of OP and osteoporotic fractures, highlighting the potential utility of serum Mg as an auxiliary biomarker for risk stratification in postmenopausal women.

## Data Availability

The raw data supporting the conclusions of this article will be made available by the authors, without undue reservation.
